# Synergetic Effects of PARP Inhibitor AZD2281 and Cisplatin in Oral Squamous Cell Carcinoma *in Vitro* and *in Vivo*

**DOI:** 10.3390/ijms17030272

**Published:** 2016-02-24

**Authors:** Masaaki Yasukawa, Hisako Fujihara, Hiroaki Fujimori, Koji Kawaguchi, Hiroyuki Yamada, Ryoko Nakayama, Nanami Yamamoto, Yuta Kishi, Yoshiki Hamada, Mitsuko Masutani

**Affiliations:** 1 Department of Oral and Maxillofacial Surgery, School of Dental Medicine, Tsurumi University, 2-1-3 Tsurumi, Tsurumi-ku, Yokohama, Kanagawa 230-8501, Japan; yasukawa-masaaki@tsurumi-u.ac.jp (M.Y.); kawaguchi-k@tsurumi-u.ac.jp (K.K.); yamamoto-nanami@tsurumi-u.ac.jp (N.Y.); kishi-y@tsurumi-u.ac.jp (Y.K.); hamada-y@tsurumi-u.ac.jp (Y.H.); 2 Department of Oral Hygiene, Tsurumi Junior College, 2-1-3 Tsurumi, Tsurumi-ku, Yokohama, Kanagawa 230-8501, Japan; 3 Division of Chemotherapy and Translational Research, National Cancer Center Research Institute, 5-1-1 Tsukiji, Chuo-ku, Tokyo 104-0045, Japan; hisakuma@ncc.go.jp (H.F.); mmasutan@ncc.go.jp (M.M.); 4 Department of Frontier Life Science, Graduate School of Biochemical Science, Nagasaki University, 1-7-1 Sakamoto, Nagasaki 852-8588, Japan; mmasutan@nagasaki-u.ac.jp; 5 Division of Maxillofacial Surgery, Department of Oral and Maxillofacial Surgery, School of Dentistry, Iwate Medical University 19-1 Uchimaru, Morioka Iwate 020-8050, Japan; yamadah@iwate-med.ac.jp; 6 Department of Pathology, School of Dental Medicine, Tsurumi University, 2-1-3 Tsurumi, Tsurumi-ku, Yokohama, Kanagawa 230-8501, Japan; nakayama-r@tsurumi-u.ac.jp

**Keywords:** PARP inhibitor, oral cancer, xenografted tumor, cisplatin

## Abstract

Cisplatin is a commonly used chemotherapeutic drug for treatment of oral carcinoma, and combinatorial effects are expected to exert greater therapeutic efficacy compared with monotherapy. Poly(ADP-ribosyl)ation is reported to be involved in a variety of cellular processes, such as DNA repair, cell death, telomere regulation, and genomic stability. Based on these properties, poly(ADP-ribose) polymerase (PARP) inhibitors are used for treatment of cancers, such as *BRCA1/2* mutated breast and ovarian cancers, or certain solid cancers in combination with anti-cancer drugs. However, the effects on oral cancer have not been fully evaluated. In this study, we examined the effects of PARP inhibitor on the survival of human oral cancer cells *in vitro* and xenografted tumors derived from human oral cancer cells *in vivo*. *In vitro* effects were assessed by microculture tetrazolium and survival assays. The PARP inhibitor AZD2281 (olaparib) showed synergetic effects with cisplatin in a dose-dependent manner. Combinatorial treatment with cisplatin and AZD2281 significantly inhibited xenografted tumor growth compared with single treatment of cisplatin or AZD2281. Histopathological analysis revealed that cisplatin and AZD2281 increased TUNEL-positive cells and decreased Ki67- and CD31-positive cells. These results suggest that PARP inhibitors have the potential to improve therapeutic strategies for oral cancer.

## 1. Introduction

The incidence of oral cancer is 1%–2% of all cancers, and an estimated 300,000 new cases were diagnosed and 145,000 patients died from oral cavity cancer (including lip cancer) in 2012 worldwide [1]. The overall incidence of oral cancer has significantly decreased over the past several decades, reflecting decreases in the consumption of tobacco and alcohol despite increased oral cancer incidence associated with oncogenic human papilloma virus [2,3]. In Japan, the incidence of cancer in oral cavity and pharynx is reported to be 7.2 out of 100,000 people (approximately 2% of all cancers) and it has been slightly increasing according to the cancer registries database [4]. Current strategies for oral cancer therapy include three major treatments: surgery, chemotherapy, radiotherapy, and their combinations [5]. The strategies for oral cancer therapy in Tsurumi University Hospital is generally decided based on NCCN (National Comprehensive Cancer Network) guidelines version 2, 2013 [6]. The first choice for primary treatment is always surgery, then, chemotherapy using cisplatin and fluorouracil or radiotherapy, or a combination of them, is chosen for adjuvant therapy. In recent years, new diagnosis techniques and treatments of oral cancer have been developed, however overall survival has not significantly decreased in the past three decades. The reason is considered to be alterations in tumor suppressor genes and changes in signaling pathways, leading to therapeutic resistance [7]. To solve these problems, studies have focused on new therapeutic strategies, including combinatorial drug regimens for cancer treatment. Such treatments are expected to exert synergetic effects resulting in greater therapeutic effects than single drug administration, decreased side-effects, and prevention of drug resistance. Based on these concepts, molecular-targeted drugs for cancer are of current research interest. For oral cancer therapy, cetuximab, which targets the epidermal growth factor receptor (EGFR) is available for clinical use. Cetuximab is activated by the ligands of EGF (epidermal growth factor) and transforming growth factor-α, and plays a crucial role in the growth and survival of many types of human cancers [8]. However, the overall response rate of oral cancer to cetuximab remains 36% [9].

On the other hand, poly(ADP-ribose) polymerase (PARP) inhibitors are another type of molecular-targeted drug [10]. They have been evaluated for (1) single treatment of breast cancer with mutations of the *BRCA1/2* gene that encodes protein involved in homologous recombination (HR) repair [11,12]; and (2) combinatorial treatments with radiotherapy or conventional chemotherapy [11,12,13]. PARP-1 is an important enzyme for base excision repair (BER) [14], and loss of PARP activity indirectly promotes accumulation of DNA double-strand breaks [15]. Therefore, *BRCA1/2*-mutated cancers treated with PARP inhibitor show synthetic lethality by the severe defect in DNA repair. PARP inhibitors are also reported to enhance the cytotoxicity of conventional chemotherapy, such as cisplatin in breast and ovarian cancers [13]. *In vitro* assessments were also reported using lymphoma, prostate cancer, and glioblastoma cells [16]. The mechanism of cisplatin is its binding to DNA and causing inter- and intra-strand cross-links, leading to DNA template defects and arrest of DNA synthesis and replication, especially in cancer cells [17]. Although the combination of cisplatin and PARP inhibitors has been evaluated in several types of cancer cells [18,19], to the best of our knowledge, it has not been evaluated in cells derived from oral cancers *in vitro* or *in vivo*.

In this study, we show that PARP-1 inhibitor AZD2281 has synergetic effects with cisplatin *in vitro* and enhances suppressive effects against the growth of xenografted tumors *in vivo*. Our results would provide a favorable possibility of administration of PARP inhibitor for oral cancer therapy in addition to conventional chemotherapy.

## 2. Results

### 2.1. Evaluation of the Cytotoxicity of the Poly(ADP-Ribose) Polymerase (PARP)-1 Inhibitor AZD2281

To investigate the cytotoxic effects of AZD2281 on three cell lines (HSC-2, Ca9-22, and SAS) derived from oral squamous cell carcinoma, we performed 3-(4,5-dimethyl-2-thiazolyl)-2,5-diphenyltetrazolium bromide (MTT) assays. The half maximal inhibitory concentration (IC_50_) of AZD2281 for HSC-2, Ca9-22 and SAS cells after 24 h of treatment was estimated to be 7.2, 15, and >20 µM, respectively (Figure S1). Next, synergetic effects of AZD2281 and cisplatin were assessed based on the results of MTT assays. Cell viability was significantly reduced by cisplatin treatment in a dose-dependent manner. The IC_50_ of cisplatin was 4.8 µM in HSC-2 cells, 9.1 µM in Ca9-22 cells, and 16.0 µM in SAS cells. With addition of 1 µM AZD2281, the IC_50_ of cisplatin was 2.4, 4.4, and 8.8 µM in HSC-2, Ca9-22, SAS cells, respectively (Figure S2A–C). The combination index (CI) of each cell line of 50% was 0.524 for HSC-2 cells, 0.549 for Ca9-22 cells, and 0.596 for SAS cells. Each CI was less than 1.0, indicating that AZD2281 had synergetic effects with cisplatin.

### 2.2. Relative Survival Assay

**Figure 1:**
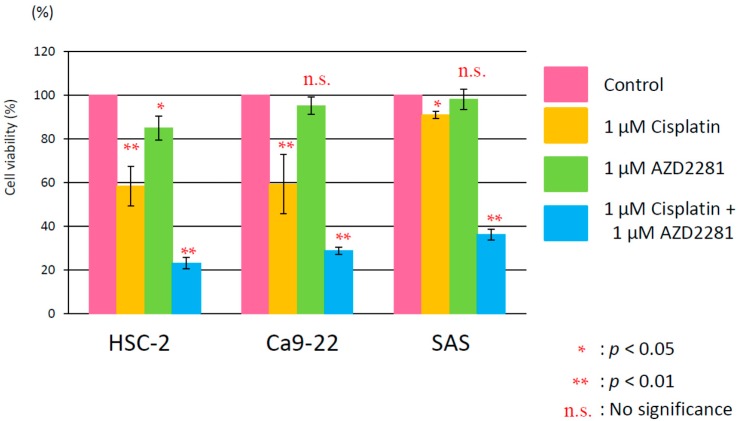
Attenuated relative cell growth by combinatorial treatment with 1 µM cisplatin and 1 µM AZD2281. Values are expressed as the mean ± SEM. * *p* < 0.05; ** *p* < 0.01; n.s., no significance.

In survival assays, combinatorial treatment with 1 or 3 µM AZD2281 and cisplatin showed significantly higher cytotoxicity than single cisplatin treatment in all three cancer cell lines (Figure S3A–C). A higher dose range of cisplatin was found to be cytotoxic. Concentrations of cisplatin capable of suppressing cell survival by 90% in the presence of 0, 1 or 3 µM AZD2281 were approximately 2.1, 1.5, and 1.2 µM in HSC-2 cells, 1.7, 1.4, and 0.98 µM in Ca9-22 cells, and 2.4, 1.5 and 1.3 µM in SAS cells, respectively. The combination of 1 µM cisplatin and 1 µM AZD2281 attenuated growth rates compared with single treatments of either cisplatin or AZD2281 (Figure 1). The results of MTT and survival assays suggested that AZD2281 showed synergetic effects with cisplatin in cell lines derived from oral squamous cell carcinoma.

### 2.3. Effects of AZD2281 and Cisplatin on Cell Cycle

**Figure 2:**
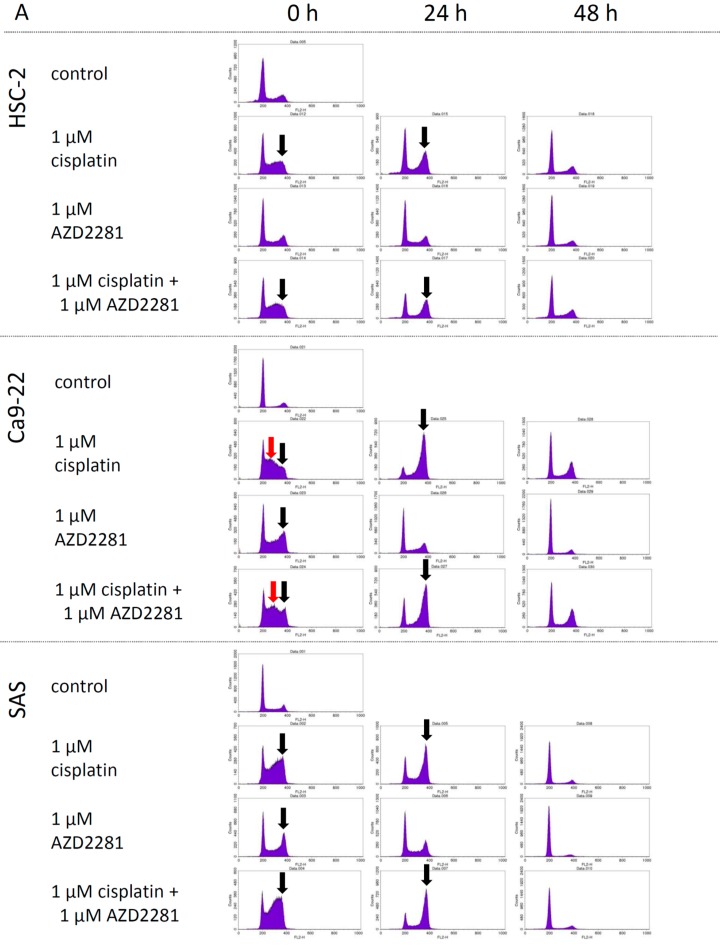

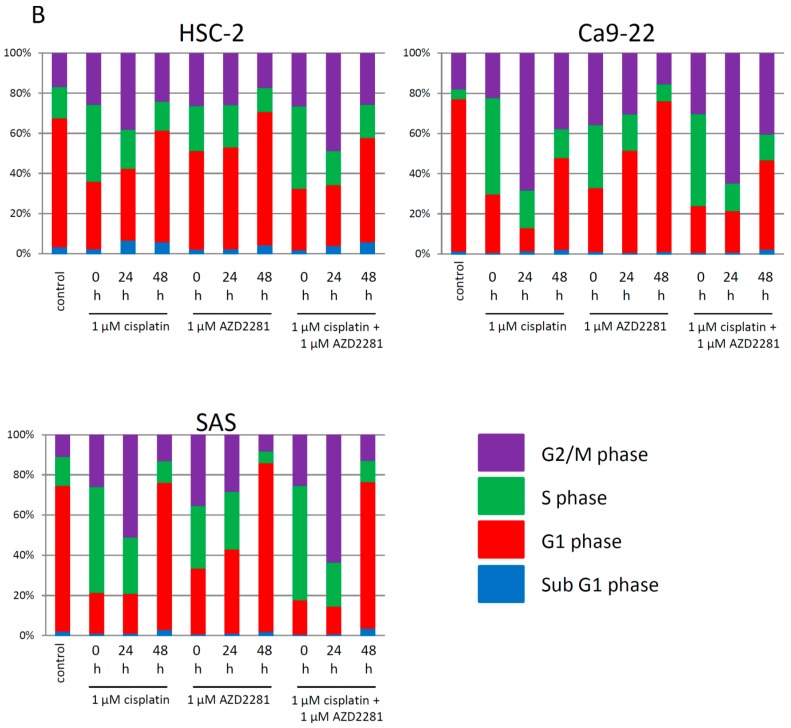
Flow cytometry analysis with propitium iodide after treatment with 1 µM cisplatin, 1 µM AZD2281, and combinatorial administration. The black arrows indicate G2/M arrest and the red arrows indicate S phase arrest (**A**); and percent distributions of cells in each sub G1, G1, S, and G2/M phase in each cell lines (**B**).

In cell cycle analysis, cells were treated with 1 µM cisplatin, 1 µM AZD2281 and their combination for 18 h and allowed to grow for 0, 24, and 48 h and analyzed. At 0 h analysis, G2/M arrest was observed in the cisplatin and the combination group of HSC-2 and SAS cell lines, and both G2/M and S phase arrest was observed in the cisplatin and the combination group of Ca9-22 cell line. Twenty-four hour after incubation, G2/M arrest was still observed in the same administration group in all cell lines, and each cell cycle was almost recovered after 48 h incubation. In all cell lines, 1 µM AZD2281 showed slight effects on cell cycle and after 24 h incubation, the cell cycle was almost recovered in all cell lines (Figure 2A). The population of G1 phase in the control group was 63.95%, 75.75%, and 72.51% in HSC-2, Ca9-22, and SAS cell lines, respectively. After cisplatin and combination drug administration, each G1 population was dramatically decreased, and recovered after 24 and 48 h incubabation. The population of sub G1 was relatively high in HSC-2 cell lines (3.53% in control group) compared to another two cell lines (Figure 2B).

### 2.4. In Vivo Effects of AZD2281 with Cisplatin on Xenografted Tumor Growth

**Figure 3:**
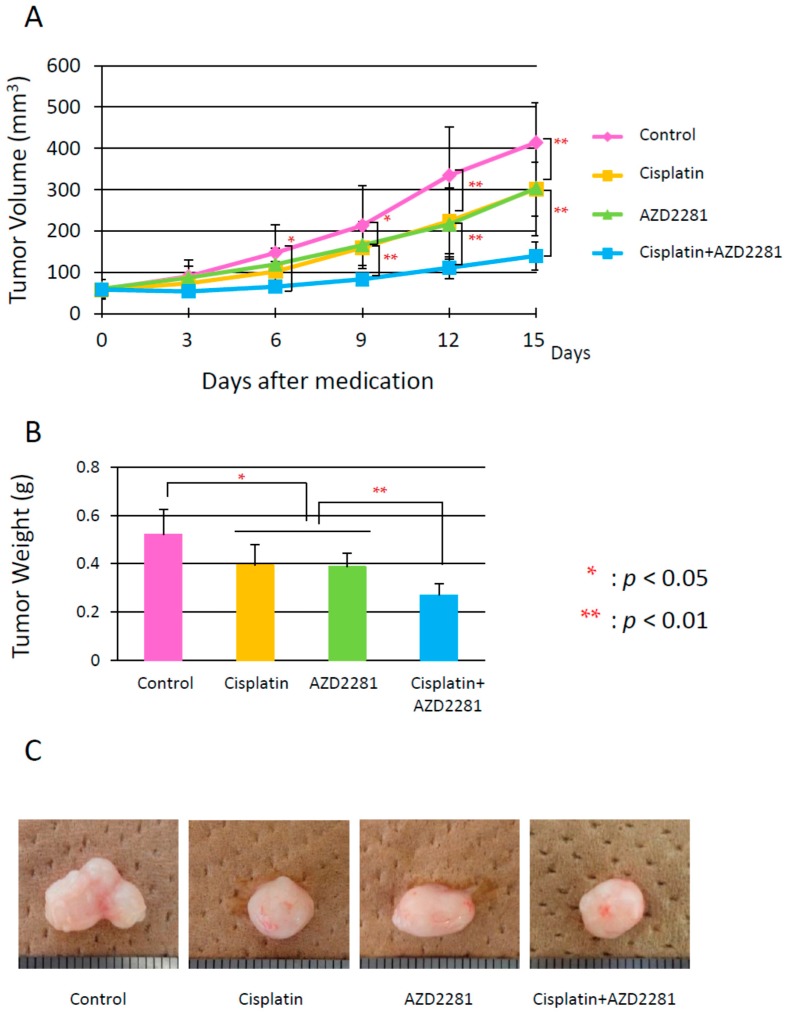
(**A**) Increases in tumor volumes during treatment with cisplatin or AZD2281, or combinatorial treatment with both cisplatin and AZD2281; (**B**) tumor weights just after sacrifice in the control, cisplatin, AZD2281, and combination groups; and (**C**) photos of representative tumors in the control, cisplatin, AZD2281, and combination treatment groups. Values are expressed as the mean ± SEM. * *p* < 0.05; ** *p* < 0.01.

Xenografted tumors were generated by subcutaneous injection of tumor cells (5 × 10^6^ cells) into the dorsal skin. Only HSC-2 cells could stably generate tumors among the used oral carcinoma cell lines. Tumor volumes of control group mice increased during the experimental period. The tumor growth of cisplatin and AZD2281 groups significantly decreased compared to the control group, and that of combination group was further decreased (Figure 3A). Cisplatin and AZD2281 groups showed almost same levels of tumor growth. After five treatments every three days, average tumor weights were 0.52, 0.39, 0.38, and 0.27 g in control, cisplatin, AZD2281, and combination groups, respectively (Figure 3B,C). Thus, AZD2281 treatment (25 mg/kg/day, every three days for five treatments) with cisplatin was considered to be effective for inhibitory growth of tumors derived from HSC-2 cells *in vivo*.

Their body weights were not significantly different both before and after the treatment (Figure S4), thus confirming that the used treatment protocol was tolerable.

### 2.5. Histopathological Analysis Reveals that the Combination of Cisplatin and AZD2281 Decreases Proliferation Potential and Increases Necrosis in Tumors

**Figure 4:**
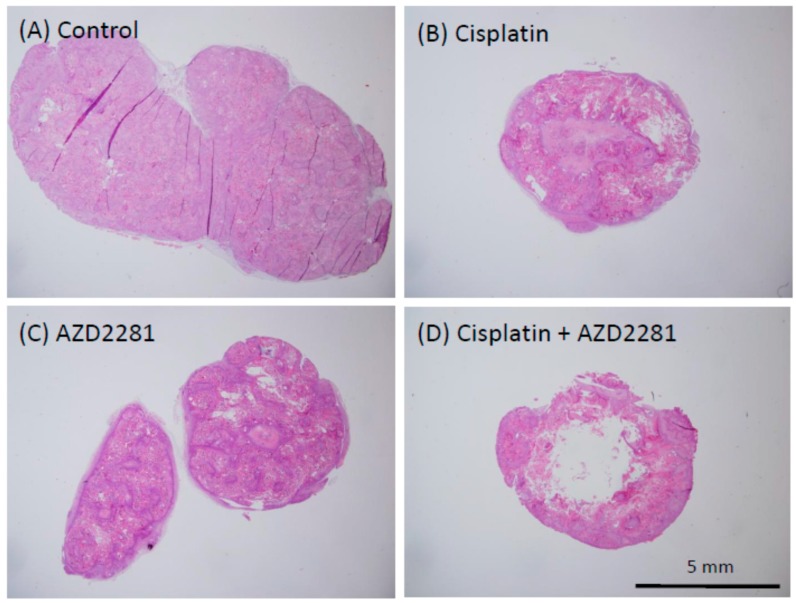
Histopathological analysis of representative xenografted tumors in control (**A**); cisplatin (**B**); AZD2281 (**C**); and combination treatment (**D**) groups. Scale bar, 5 mm.

**Figure 5:**
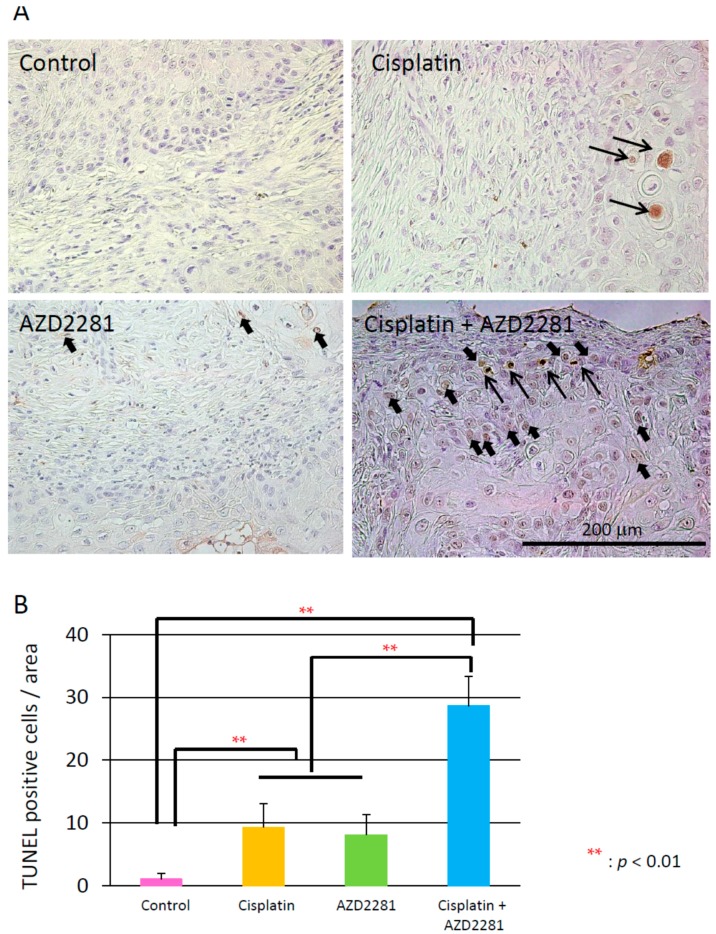
(**A**) TUNEL staining of xenografted tumors in control (**upper left**); cisplatin (**upper right**); AZD2281 (**lower left**); and combination (**lower right**) groups. Open arrows indicate apoptotic cells, and closed arrows indicate pre-apoptotic cells. Scale bar, 200 µm; (**B**) TUNEL-positive cells (both apoptotic and pre-apoptotic cells) per area in each treatment group. ** *p* < 0.01.

**Figure 6:**
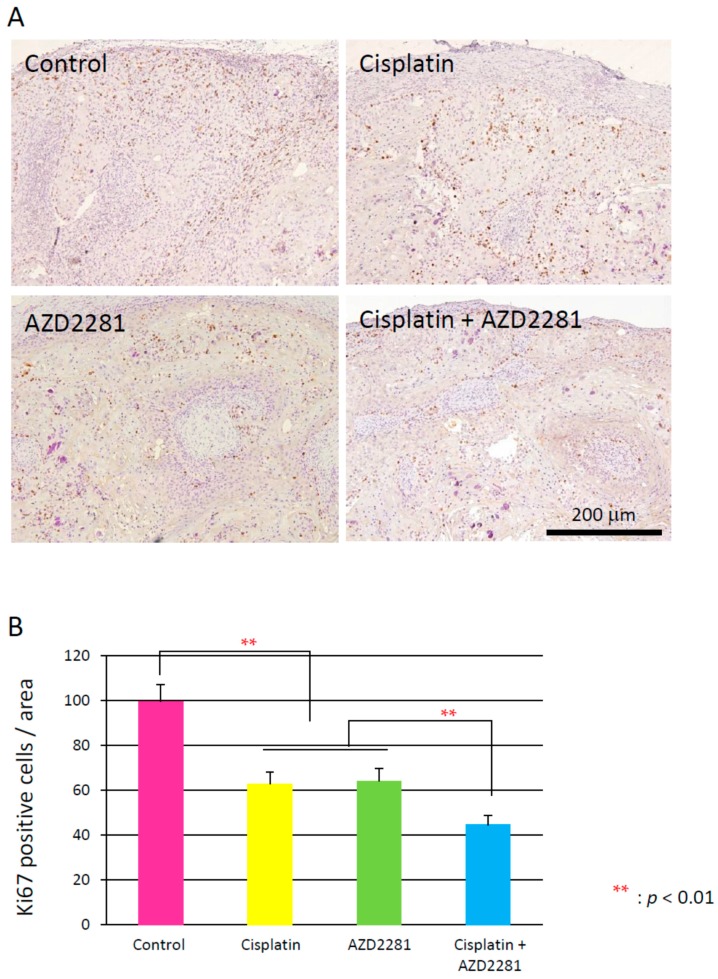
(**A**) Immunohistochemical analysis of Ki67 in xenografted tumors of control (**upper left**); cisplatin (**upper right**); AZD2281 (**lower left**); and combination (**lower right**) groups. Scale bar, 200 µm; (**B**) Ki67-positive cells per area in each treatment group. ** *p* < 0.01.

**Figure 7:**
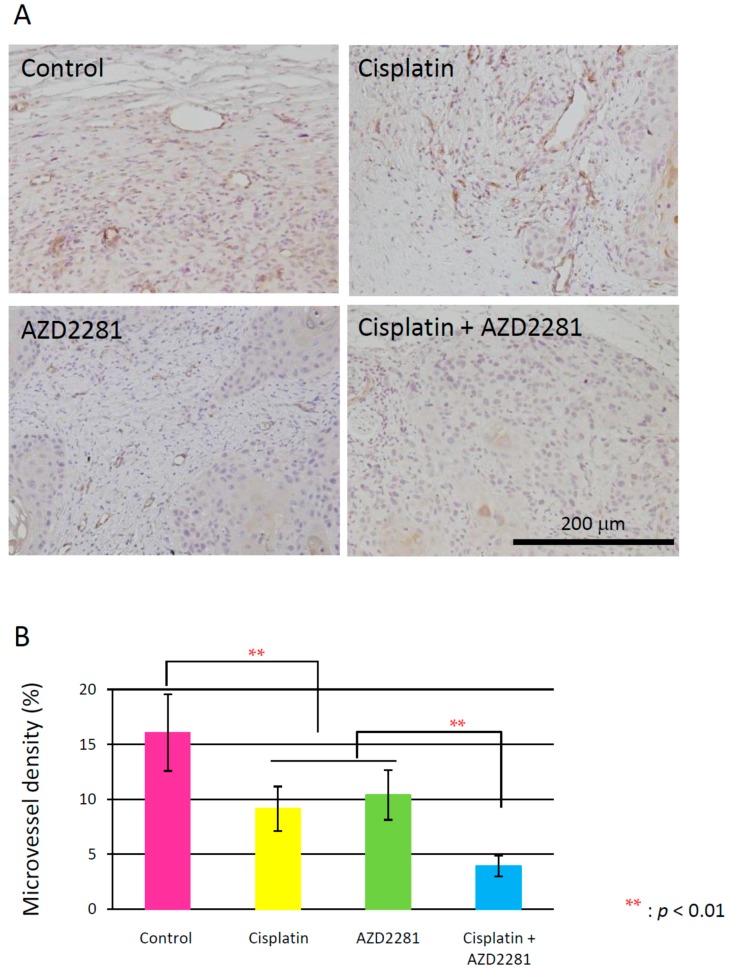
(**A**) Immunohistochemical analysis of CD31 in xenografted tumors of the control (**upper left**); cisplatin (**upper right**); AZD2281 (**lower left**); and combination (**lower right**) groups. Scale bar, 200 µm; (**B**) CD31-positive cells per area in each treatment group. ** *p* < 0.01.

Microscopic analysis of hematoxylin and eosin-stained HSC-2 tumors of the three treatment groups showed hollowed out areas caused by necrosis compared to the control group. The center of tumors in the combination group showed larger necrotic hollowed area, while tumors in cisplatin and AZD2281 groups showed scattered hollowed areas (Figure 4). Coagulative necrosis by chemotherapy was also observed outward from the center in the cisplatin group and partially in the AZD2281 group. In the combination group, stronger coagulative necrosis was observed in addition to keratinizing degeneration (Figure S5). Terminal deoxynucleotidyl transferase (TdT) dUTP (2′-deoxyuridine 5′-triphosphate) nick-end labeling (TUNEL) assays showed that tumors of the combination group had significantly more TUNEL-positive cells, and both apoptotic and pre-apoptotic cells were observed (Figure 5). In the control group, only a few TUNEL-positive apoptotic cells were observed by microscopy, while strong apoptosis was mainly observed in tumor cells in the cisplatin group and pre-apoptotic cells were observed in small nuclear stroma cells in the AZD2281 group. In combination group, both cell types showed TUNEL-positivity. To evaluate the therapeutic effect of the combination of cisplatin and AZD2281, tumor cell proliferation ability and microvessel density were also measured. Ki-67 expression was widely observed in tumors of the control group, while it was significantly reduced in cisplatin and AZD2281 groups to 62.7% (*p* < 0.05) and 63.9% (*p* < 0.05), respectively. Consistent with the higher sensitivity to the combination of cisplatin and AZD2281, Ki-67 expression was decreased by combinatorial treatment to 44.5% of the control group (*p* < 0.01) (Figure 6). A similar tendency was also observed in the tumor microvessel density when CD31 expression was evaluated. CD31 expression was strongly positive in the control group, however it was significantly reduced in cisplatin and AZD2281 groups to 56.8% (*p* < 0.01) and 64.7% (*p* < 0.01), respectively. Moreover, CD31 expression was further decreased by the combinatorial treatment to 24.5% (*p* < 0.01). Small vessel formation was hardly observed in the combination group, while it was observed in the other three groups (Figure 7). These results suggested that decreased proliferation potential, increased necrosis and apoptosis, and reduction in vascular formation might be the causes of the slower tumor growth *in vivo*.

### 2.6. Results of Analysis of Western Blotting

**Figure 8:**
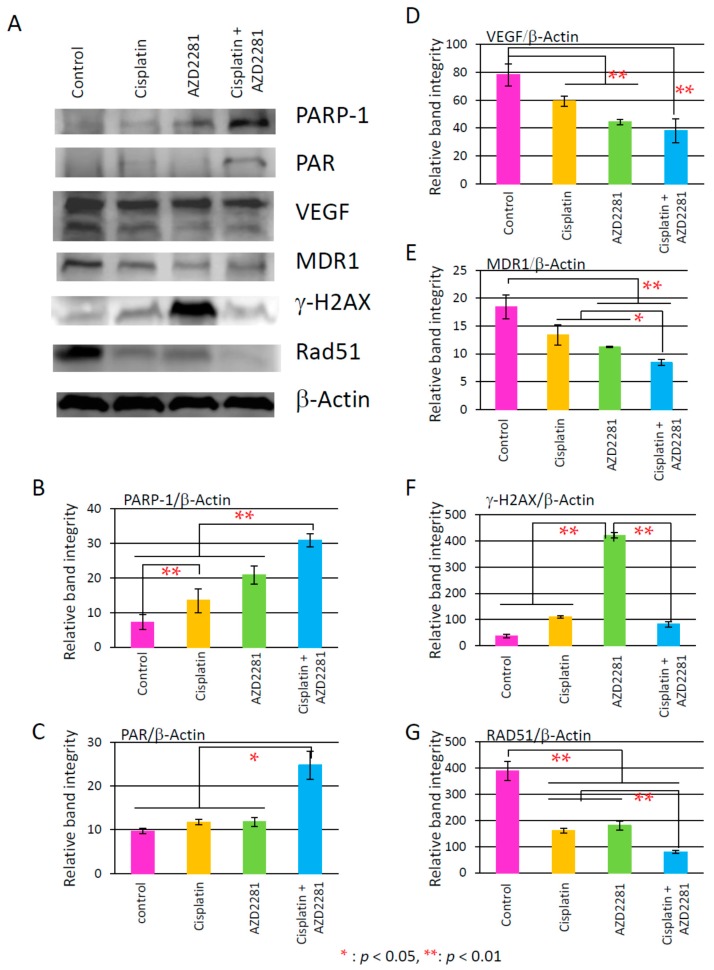
(**A**) The effect of cisplatin and AZD2281 on the protein expression of xenografted HSC-2 tumors. Relative band intensity of western blot data was normalized by the expression level of β-actin; The proteins analyzed were PARP-1 (poly(ADP-ribose) polymerase-1) (**B**); poly(ADP-ribose) (PAR) (**C**); vascular endotherial growth factor (VEGF) (**D**); multi-drug resistance gene 1 (*MDR1*) (**E**); *γ-H2AX* (**F**); and *RAD51* (**G**). Values are expressed as the mean ± SEM. * *p* < 0.05; ** *p* < 0.01.

To assess the mechanism of the synergetic effects of cisplatin and AZD2281, we analyzed the protein levels of factors related to poly(ADP-ribosyl)ation, vascular endothecial growth factor (VEGF), multi-drug resistant gene 1 (*MDR1*), γ-H2AX, and *RAD51* after HSC-2-derived tumors were resected on day 18 of medication. Protein levels of PARP-1 and poly(ADP-ribose) (PAR) were significantly increased in the combination group compared to the control, and those of VEGF and MDR1 were significantly reduced in the combination group, which was consistent with inhibition of tumor growth *in vivo*. RAD51 expression was significantly reduced in the cisplatin and AZD2281 groups, and further in the combination groups, possibly leading to attenuation of HR. The γ-H2AX level was significantly increased only in the AZD2281 group, suggesting the significant increase of double-strand breaks in this condition (Figure 8).

## 3. Discussion

In this study, the PARP-1 inhibitor AZD2281 showed *in vitro* synergetic effects in combination with cisplatin in three cell lines derived from oral carcinoma, HSC-2, Ca9-22 and SAS, and partly *in vivo* synergetic effects in xenografted HSC-2 tumors.

Enhanced anti-tumor effects of PARP inhibitors with cisplatin have been reported, especially in breast cancer [20,21,22]. Furthermore, the combination of PARP inhibitors with temozolomide in Ewing’s sarcoma [23] or camptothecin in childhood neuroblastoma [24] shows stronger synergetic effects compared to the cisplatin case. In a previous report, cell viability curves were compared between temozolomide, camptothecin, and cisplatin in DT40 cells (chicken lymphoma cell line) and DU145 cells (prostate cancer cell line), and PARP inhibitor just added its own cytotoxicity [16]. Compared to previous reports, viability curve of our data showed synergetic effects (Figures S2 and S3). Moreover, the CI of our data was around 0.55, which was relatively higher than that of DT40 cells and much lower than that of DU145 cells. Based on our data and previous studies, the CI could be quite different among cell types.

The synergetic effects of AZD2281 and cisplatin could be related to cell cycle. AZD2281 had been reported to cause G2/M arrest and decrease DNA repair activity through G2 cell-cycle arrest-like effect in a dose and p53-dependent manner [25], and cisplatin also causes G2/M arrest [26]. Our data also showed that the increased G2/M arrest in the combination group could be involved in the synergetic effects. Moreover, the cell cycle arrest profiles, namely the highest apoptotic sub-G1 population in the HSC-2 cell line and S-phase arrest in the Ca9-22 cell line, might contribute to the difference in the sensitivity and cytotoxicity within the three cell lines [27].

*In vivo*, xenografted HSC-2 tumors also showed the similar synergetic effect after combinatorial treatment with cisplatin and AZD2281. Although the reduction rate of xenografted tumor volumes was almost additive (Figure 3A–C), central necrosis, strong TUNEL-positive staining, and reduced numbers of Ki67-positive cells, which were observed in the combination group, were consistent with the results obtained from *in vitro* experiments. The expression level of RAD51 was significantly decreased in the cisplatin and AZD2281 groups, and even more in the combination group. This could lead to deficient HR pathway by the combinatorial treatment. Moreover, PARP-1 activation and synthesis of PAR were much higher in the combination group than other three groups. Consequently, the level of DNA damage could be highest in the combination group although γ-H2AX expression level was highest in AZD2281 group.

The mechanism of DNA damage caused by cisplatin is the interaction with DNA to form DNA adducts, primarily intra-strand crosslink adducts, while the mechanisms of drug resistance could involve limited DNA damage due to reduction of drug uptake and increase of drug inactivation and DNA adduct repair. These platinum-DNA adducts’ removal and repair of DNA damage are mediated mainly by nucleotide excision repair (NER) and HR [28]. PARP-1 is mainly involved in base excision repair (BER). There are several reports of PARP-1 involvement in NER. For example, PARP-1 collaborates with DDB2 (damaged DNA-binding protein 2) to increase the efficiency of the lesion recognition step of global genomic NER [29]. In addition, PARP-1 regulates XPA (xeroderma pigmentosum complementation group A protein), which is a key protein of NER [30]. Therefore, the synergetic effects in the combination group might be related to a decrease in the interactions of PARP-1 with DDB2 or XPA, resulting in the increased sensitivity to cisplatin. Of note, our results also showed that the protein level of MDR1 was significantly decreased in the combination group (Figure 8E). Therefore, the development of drug resistance might be decreased by the combinatorial treatment with AZD2281 although the mechanism was not elucidated.

On the other hand, angiogenesis-related factors, such as CD31 and VEGF, were also attenuated after the combinatorial treatment with AZD2281 and cisplatin, suggesting that AZD2281 is also involved in inhibition of the angiogenesis pathway. The relationship has been previously reported between poly(ADP-ribosyl)ation and CD31 and VEGF expression in vascular smooth muscle cells (VSMCs) [31,32], such as facilitation of DNA repair by prolonged poly(ADP-ribosyl)ation in VSMCs, and the direct effect of PARP inhibitor on tumor vascularization through changes in CD31 expression levels was reported in *in vivo* models of anaplastic thyroid carcinoma.

Considering our results and previous studies, synergetic effects of cisplatin and AZD2281 could be caused by (1) cisplatin effects that cause inter- and intra-strand crosslinks; (2) AZD2281 effects that attenuate BER; (3) their possible synergetic effects caused through deficient NER and HR; and (4) a possible direct attenuation of tumor vascularization by AZD2281. Therefore, considering the therapeutic potential of PARP inhibitors in the treatment of oral cancer, the current study suggested that combination of cisplatin and AZD2281 is one of the promising candidates for treatment strategy. Our *in vivo* experiments were performed using only one cell line, therefore further investigations of the mechanism responsible for the suppression of HR and NER pathways, or side-effects in combination with cisplatin and PARP inhibition will be necessary. Meanwhile, it would provide new insights into cancer therapies.

## 4. Materials and Methods

All animal experimental procedures were approved by the ethical committee for the guidelines on animal experiments of Tsurumi University School of Dental Medicine on 3 July 2012 (No. 12040).

### 4.1. Culture of Cell Lines Derived from Oral Carcinoma

Three cell lines, HSC-2 (human oral squamous cell carcinoma cell line), Ca9-22 (human gingival carcinoma cell line), and SAS (human tongue squamous cell carcinoma cell line) (all obtained from RIKEN, Tsukuba, Japan, on 11 October 2012) were used in this study. Growth medium consisted of 10% fetal bovine serum (Biowest, Nuaillé, France), 100 U/mL penicillin, and 100 µg/mL streptomycin (Sigma-Aldrich, St. Louis, MO, USA) in minimum essential Eagle’s medium (Sigma-Aldrich) for HSC-2 and Ca9-22 cells, and in Dulbecco’s Modified Eagle’s Medium (Sigma-Aldrich) for SAS cells. The cells were maintained at 37 °C with 5% CO_2_. Growth medium was changed every three days. Cells were passaged at 1:5 after it reached confluence.

### 4.2. MTT (3-(4,5-Dimethyl-2-thiazolyl)-2,5-diphenyltetrazolium bromide) Assay

HSC-2, Ca9-22, and SAS oral carcinoma cells were seeded in 24-well plates at a density of 2 × 10^4^ cells/well. After overnight incubation, the culture medium was replaced with fresh medium containing various concentrations of PARP inhibitor AZD2281 (ChemScene, Monmouth Junction, NJ, USA) or cisplatin (*cis*-diammineplatinum(II) dichloride, Sigma-Aldrich). After 24 h of treatment, the number of viable cells was assessed using an MTT assay as reported previously [33]. Briefly, one tenth of the fluid volume of 5 mg/mL MTT (3-(4,5-dimethylthiazol-2-yl)-2,5-diphenyltetrazolium bromide) in RPMI-1640 medium (Sigma-Aldrich) was added to each well, followed by incubation for 4 h at 37 °C. After incubation, the medium was carefully removed and an adequate volume of 0.1 N HCl (Wako Pure Chemical Industries, Ltd., Osaka, Japan) in isopropanol (Wako Pure Chemical Industries, Ltd.) was added to each well and the resultant formazan crystals was dissolved. Absorbance was determined at 570 nm by microplate reader (Model 680; Bio-Rad, Hercules, CA, USA) in 96-well assay plates (Sumilon, Sumitomo Bakelite, Tokyo, Japan). All experiments were performed in triplicate.

IC_50_ was calculated by Excel software (Microsoft, Redmond, Washington, DC, USA) using the logarithm function. First, the concentration was plotted on the *x*-axis, and cell viability was plotted on the *y*-axis. Then, using the value of higher and lower sides of the 50% concentration and cell viability, a linear equation was created as follows:

$${\text{IC}}_{50}=10^{\log(A/B)\times (50-C)/(D-C)+\log B}$$

where *A* is the concentration of the higher side of 50% cell viability, *B* is the concentration of the lower side of 50% cell viability, *C* is the cell viability at the concentration of *B*, and *D* is cell viability at the concentration of *A*.

### 4.3. Survival Assay

2 × 10^4^ cells were seeded in each of 12-well plates and cultured in growth medium with various concentrations of cisplatin with 0, 1, or 3 µM AZD2281 for 18 h. After washing with phosphate-buffered saline (PBS), the cells were allowed to grow. Then, cell numbers were counted using a hemocytometer when cells without AZD2281 reached confluence. Suppression of cell survival by 50% was calculated using the formula as described above.

### 4.4. Combination Index Analysis

The results were analyzed using the median effect/CI isobologram equation that is based on the median effect principle (mass action law) [34,35]. First, the dose effect curves for cisplatin and AZD2281, and their combination were plotted using the following equation:

$$\log\left(\frac{fa}{fu}\right)=m\log D-m\log D_{m}$$

where *fa* is the fraction affected by dose *D*, the (%) inhibition:

$$\%\text{inhibition}=\frac{\text{absorbance of MTT assay in drug presence}}{\text{absorbance of MTT assay in drug absence}}\times 100$$

where *fu* is the uninhibited fraction (1 − *fa*), *D* is the administered drug dose (concentration), *D_m_* is the dose (concentration) required for 50% inhibition (the median effect), and *m* is defined as the coefficient of the sigmoidicity of the dose effect curve.

Next, to determine the dose of single drugs (*D_x_*) or their combination (*D*1,2)_x_ necessary to achieve x% inhibition, the median effect relationship was re-expressed as follows:

$$D_{x}=D_{m}{\left(\frac{fx}{1-fx}\right)}^{1/m}$$

where *D_x_* is the dose concentration required for *x*% inhibition.

Finally, the interaction of the two drugs was assessed by the CI as follows:

$$\text{CI}=\frac{D_{1}\text{ combined}}{D_{1}\text{ alone}}+\frac{D_{2}\text{ combined}}{D_{2}\text{ alone}}$$

where *D*_1_ combined is the dose of cisplatin in combination with AZD2281, *D*_2_ combined is the dose of AZD2281 in combination with cisplatin, *D*_1_ alone is the single dose of cisplatin required for *D_x_* with the combination of cisplatin and AZD2281, and *D*_2_ alone is the single dose of AZD2281 required for *D_x_* with the combination of cisplatin and AZD2281. CI < 1.0 = synergetic, CI = 1.0 = additive, and CI > 1.0 = antagonistic.

### 4.5. Flow Cytometry

Cells were seeded in 10 cm-diameter plates at a density of 1 × 10^5^ cells/well and cultured in growth medium with 1 µM cisplatin or 1 µM AZD2281, and their combination for 18 h. After washing with PBS, the cells were allowed to grow for 0, 24, and 48 h. Then, each supernatant was collected, cells were washed with PBS and treated with Accutase (Innovative Cell Technologies, San Diego, CA, USA) at 37 °C for 10 min. Cells were centrifuged, washed with PBS, and fixed with cold 70% ethanol for 1.5 h. Subsequently, cells were filtered with a cell strainer (Corning, Corning, NY, USA) and collected in sample tubes. Cells were then treated with 0.2 mg/mL of RNaseA and 20 µg/mL of PI (3,8-Diamino-5-(3-(diethylmethylammonio)propyl)-6-phenylphenanthridinium diiodide, Dojindo Molecular Technologies, Inc., Kumamoto, Japan) in PBS in a dark condition for 30 min. Then, cells were analyzed by FACS Calibur (Beckton and Dickinson, Franklin Lakes, NJ, USA).

### 4.6. Animal Model

Male Balb/c nu/nu mice (SLC Co., Ltd., Shizuoka, Japan) weighing about 20 g at six-weeks old each were used in this study. They were maintained under specific pathogen-free conditions throughout the experiments with constant room temperature, and a 12 h night and day cycle. The mice were continuously supplied with normal food chow (CE-2; SLC Co., Ltd.) and sterilized water *ad libitum*. They were anesthetized and HSC-2, Ca9-22, and SAS cells were injected into their dorsal skin subcutaneously (5 × 10^6^ cells in 200 µL growth medium per mouse). Within two weeks, tumors usually enlarged to the diameter of more than 7 mm. Among the three cancer cell lines, only HSC-2 cells could stably develop tumors. Therefore, *in vivo* experiments were performed using HSC-2 cell-derived xenografted tumors.

### 4.7. Animal Experimental Protocol

Once the tumor diameter had reached 7 mm, the mice were randomly assigned to the following groups: (a) control (200 µL saline); (b) cisplatin (2 mg/kg per body weight, dissolved in 200 µL sterilized water); (c) AZD2281 (25 mg/kg per body weight, dissolved in 200 µL sterilized water); or (d) combination (both cisplatin and AZD2281). The chemicals were administered intraperitoneally every three days, five times. Although AZD2281 is administered orally in the clinic, intraperitoneal injection was recommended by the manufacturer because of easier manipulation and the ethical constraints associated with oral gavage administration to mice. Tumor size and body weight were measured at the time of administration. The tumor volume was calculated using following equation [36].

Tumor volume = verticality × width × height ×0.5236

Three days after the last administration, all surviving mice were sacrificed.

### 4.8. Histopathological Analysis

Mice were treated as described above and then sacrificed. Tumors were removed and immediately fixed in 10% formalin/PBS (Wako Pure Chemical Industries, Ltd.) for 48 h, followed by immersion in 70% alcohol for 48 h and embedding in paraffin. Tissue sections (4 µm) were mounted on silane-coated slides (New Silane III, Muto Pure Chemicals Co., Ltd., Tokyo, Japan), deparaffinized with xylene (Wako Pure Chemical Industries, Ltd.), and rehydrated with graded alcohol solutions (Wako Pure Chemical Industries, Ltd.). The specimens were pathologically analyzed by hematoxylin and eosin staining (HE; Merck KGaA, Darmstadt, Germany).

### 4.9. Terminal Deoxynucleotidyl Transferase (TdT) dUTP Nick-End Labeling (TUNEL) Assay

After deparaffinization with xylene and rehydration with graded alcohol solutions, the sections were incubated in 20 µg/mL of proteinase K (Boehringer; Mannheim, Germany) dissolved in TBS (Takara, Ohtsu, Shiga, Japan) for 15 min at room temperature. Then, TUNEL assay was performed using an ApopTag^®^ Peroxidase *in Situ* Apoptosis Detection kit (Merck Millipore, Darmstadt, Germany) according to the manufacturer’s protocol. Apoptotic and necrotic cells were visualized with the Super Sensitive DAB (3,3′-diaminobenzidine) Substrate Pack (BioGenex, Fremont, CA, USA) according to the manufacturer’s protocol, followed by counterstaining with hematoxylin and mounting. An apoptotic cell was defined as a round or oval cell, dense with nuclear staining with DAB, and a pre-apoptotic cell was defined as a cell with cytoplasmic and nuclear staining with DAB, otherwise normal in cellular appearance.

### 4.10. Immunohistochemistry

After deparaffinization with xylene and rehydration with descending concentrations of ethanol, antigen retrieval was performed using Immunosaver (Nisshin EM, Tokyo, Japan) according to the manufacturer’s protocol. Briefly, sections were incubated in Immunosaver (1:200 dilution in tap water) for 40 min at 95 °C and then transferred to tap water and incubated for 10 min at room temperature. Subsequently, endogenous peroxidase was inactivated by treatment with 3% hydrogen peroxide (Wako Pure Chemical Industries, Ltd.) in methanol (Wako Pure Chemical Industries, Ltd.) for 30 min at room temperature. After treatment with 20% normal goat serum (Nichirei Corporation, Tokyo, Japan) for 30 min at room temperature, sections were incubated with the primary antibody at 4 °C overnight. Antibodies used in this study were rabbit polyclonal anti-Ki67 antibody (1:2 dilution, Dako, Troy, MI, USA) and rabbit polyclonal anti-CD31 antibody (1:50 dilution, Abcam, Cambridge, UK). Antibodies were diluted with PBS (pH 7.4) containing 1% bovine serum albumin (Sigma-Aldrich) and incubated at 4 °C. After several PBS washes, bound antibodies were visualized using Histofine Simple Stain MAX-PO(MULTI) (Nichirei Corporation) and DAB (Vector Laboratories, Inc., Burlingame, CA, USA) according to the manufacturer’s protocol. The sections were counterstained with hematoxylin and mounted. As a negative control, sections were processed without exposure to the primary antibody. The sections were viewed under a BX51 System Microscope (Olympus, Tokyo, Japan). Images were recorded using a digital microscope camera (DP70; Olympus).

### 4.11. Western Blotting

A small portion of HSC-2 xenografted tumors in each group were lysed with RIPA buffer (10 mM Tris-HCl, 1% NP-40, 0.1% SDS, 150 mM NaCl, and 1 mM EDTA) containing protease inhibitor cocktail (Thermo Fisher Scientific, Waltham, MA, USA). Each tumor was minced and centrifuged at 14,000× *g* for 20 min at 4 °C. NuPAGE LDS sample buffer (Invitrogen, Carlsbad, CA, USA) was added to the supernatant. Subsequently, the samples were heated at 98 °C for 5 min. 80 µg of protein samples were separated electrophoretically with 8% Bis-Tris gels by the NuPAGE System and subsequently electroblotted onto a polyvinylidene difluoride membrane using an iBlot Dry Blotting System (all were from Invitrogen). After blocking with 5% dry skim milk (Wako Pure Chemical Industries, Ltd.) at room temperature for 30 min, the membranes were treated with primary antibody dissolved in 2.5% dry skim milk at 4 °C overnight. The primary antibodies used in this study were anti-PARP-1 (1:200 dilution, Santa Cruz Biotechnology, Santa Cruz, CA, USA), anti-PAR (1:1000 dilution, Trevigen, Gaithersburg, MD, USA), anti-VEGF (1:500 dilution, Bioss Antibodies, Woburn, MA, USA), anti-MDR1 (1:1000 dilution, Bethyl Laboratories, Inc., Montgomery, TX, USA), anti-γ-H2AX (1:250 dilution, Abcam), and anti-RAD51 (1:500 dilution, GeneTex Inc., Irvine, CA, USA). Then, incubation of membranes was performed with peroxidase-conjugated secondary antibodies at room temperature for 30 min. Subsequently, bands were visualized by Amersham ECL Prime Western Blotting Detection Reagent (GE Healthcare, Buckinghamshire, UK). Then, images were scanned by a C-DiGit Scanner and analyzed by Image Studio Lite Software (both from Li-COR Biosciences, Lincoln, NE, USA).

### 4.12. Statistical Analysis

Group comparisons were carried out using an independent *t*-test. All data were statistically analyzed by SPSS v.20.0 (IBM Corp., Armonk, NY, USA).

## 5. Conclusions

In this study, the growth of oral carcinoma cells was attenuated by single treatments with cisplatin or AZD2281, and further attenuated by combinatorial treatment with combination of cisplatin and AZD2281 *in vitro* and *in vivo*. The *in vitro* and *in vivo* results suggest that poly(ADP-ribosyl)ation is involved in suppression of oral carcinoma growth and the effects are synergetic. Moreover, AZD2281 treatment could decrease the *MDR* gene expression, suggesting that drug resistance could be prevented by AZD2281 treatment. Taken together, our results indicate that AZD2281 would be one of a number of promising molecular-targeted drugs for oral carcinoma therapy.

## Supplementary Materials

Supplementary materials can be found at http://www.mdpi.com/1422-0067/17/3/272/s1.
