# Supplementary material for: Synergetic Effects of PARP Inhibitor AZD2281 and Cisplatin in Oral Squamous Cell Carcinoma in Vitro and in Vivo
Source: Int J Mol Sci. 2016 Feb 24;17(3):272. doi: 10.3390/ijms17030272 (PMC4813136; doi:10.3390/ijms17030272)
Supplement: Supplementary file 1 [file ijms-17-00272-s001.pdf]

Supplementary Material: Synergetic Effects of PARP Inhibitor AZD2281 and Cisplatin in Oral Squamous Cell Carcinoma *in Vitro* and *in Vivo*

Masaaki Yasukawa, Hisako Fujihara, Hiroaki Fujimori, Koji Kawaguchi, Hiroyuki Yamada, Ryoko Nakayama, Nanami Yamamoto, Yuta Kishi, Yoshiki Hamada and Mitsuko Masutani

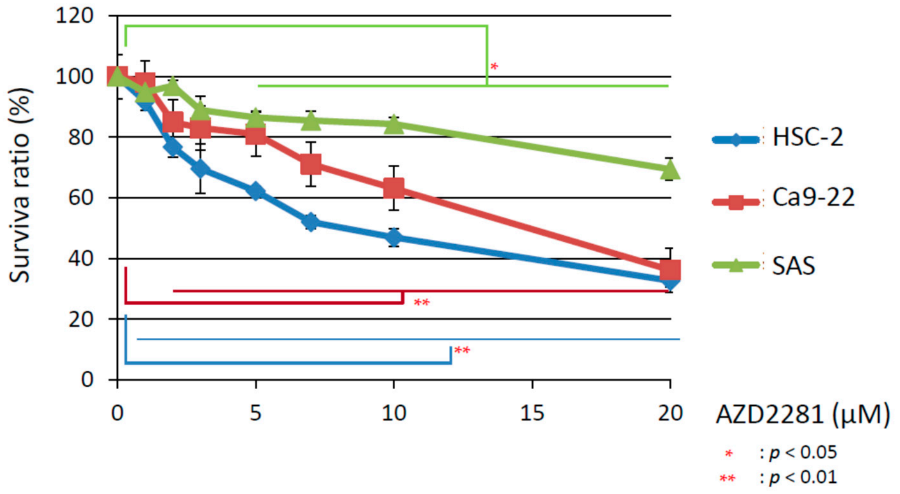

Figure S1. Result of MTT assay in three oral carcinoma cell lines. Survival ratio was decreased by the treatment with AZD2281 in a dose dependent manner in three oral carcinoma cell lines. Values are expressed as the mean ± SEM. * $p < 0.05$; ** $p < 0.01$.

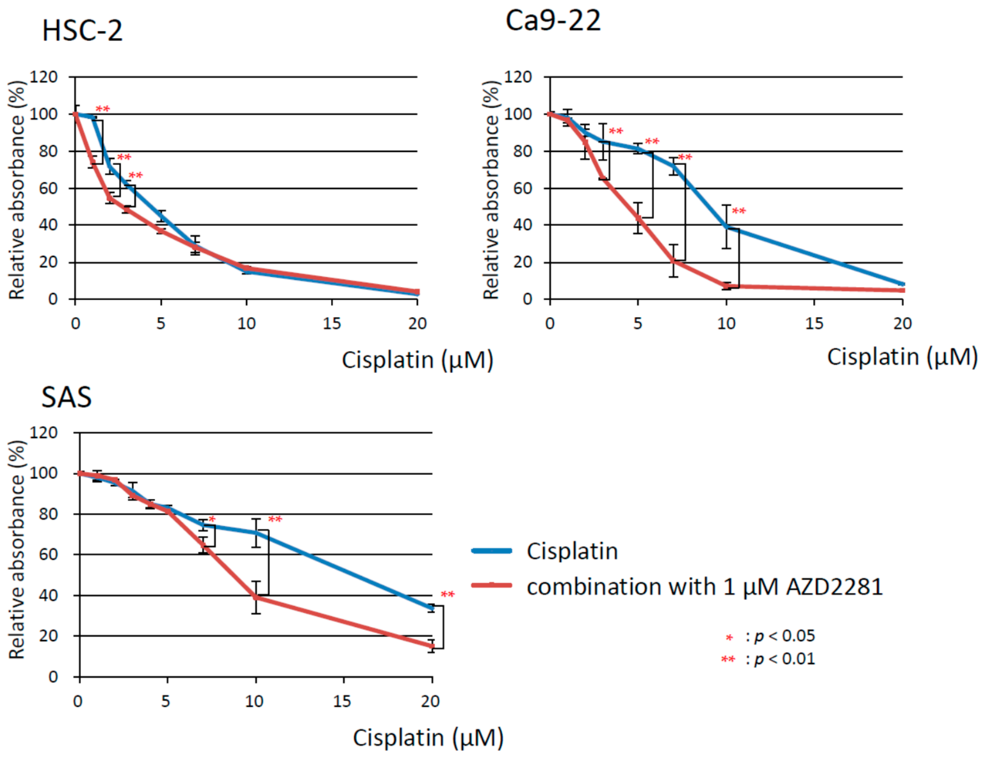

Figure S2. Results of MTT assay after combinatorial treatment of AZD2281 and cisplatin. With 1 μM AZD2281, the cytotoxicity was significantly higher compared to single treatment of cisplatin. Values are expressed as the mean ± SEM. * $p < 0.05$; ** $p < 0.01$.

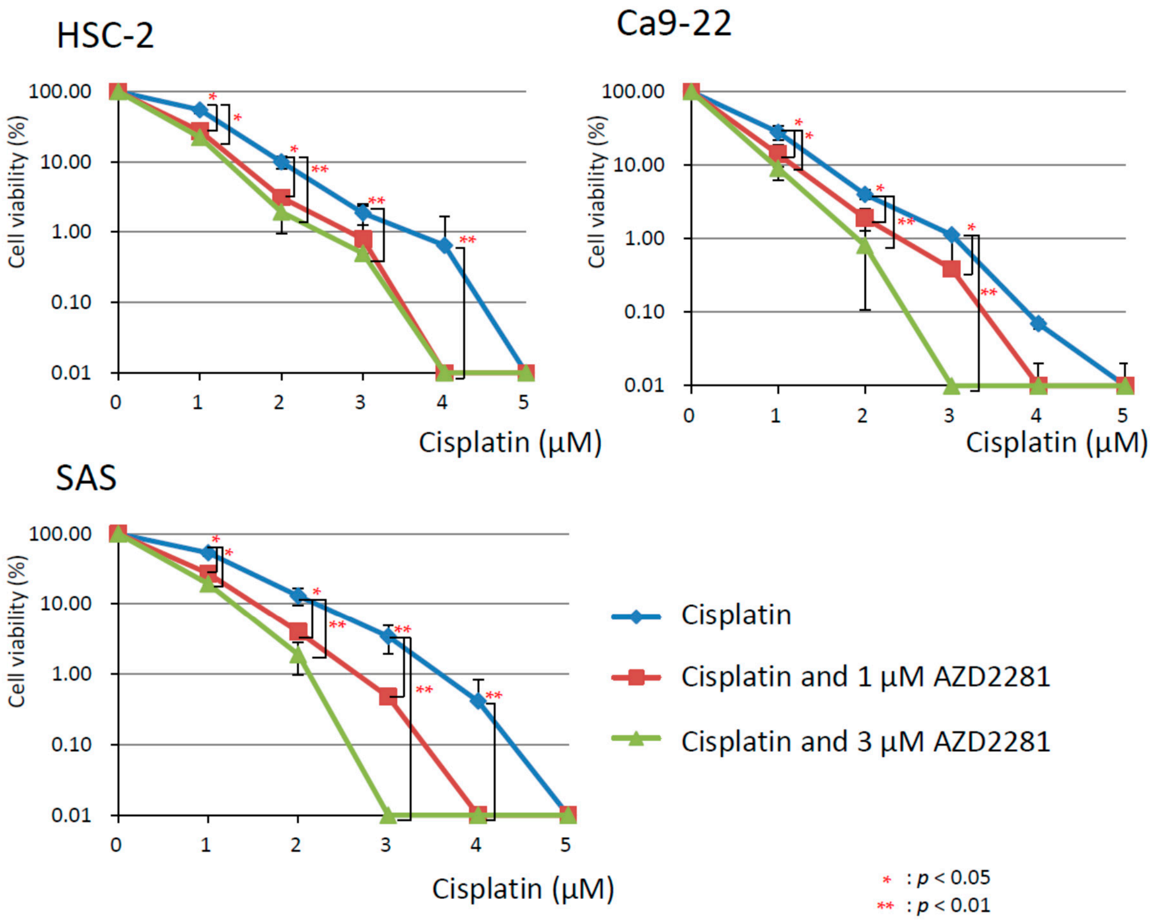

Figure S3. Results of survival assay of three oral carcinoma cell lines after treatment with cisplatin and AZD2281. Combination treatment of cisplatin and AZD2281 showed significant decrease of cell survival in each three oral carcinoma cell lines. Values are expressed as the mean ± SEM. * $p < 0.05$; ** $p < 0.01$.

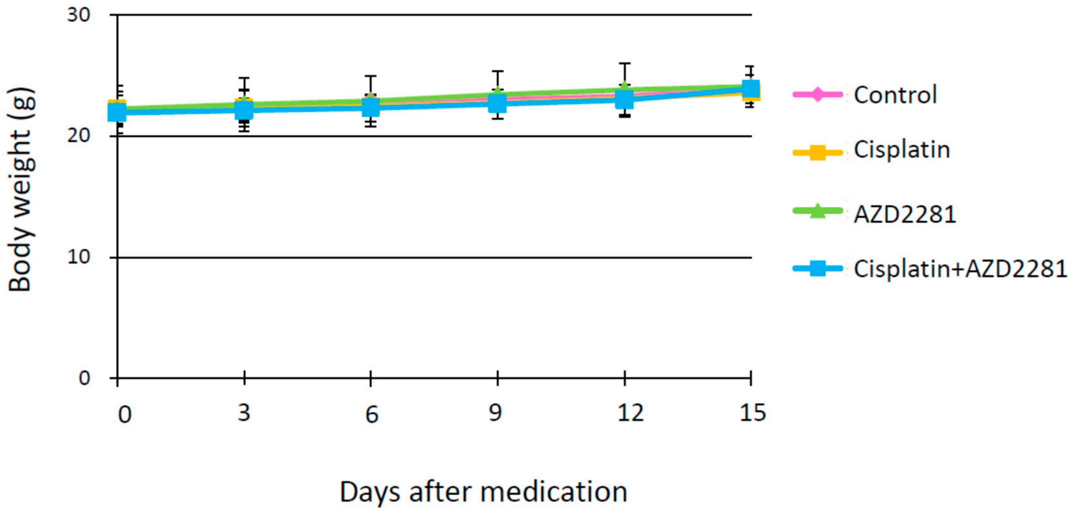

Figure S4. Body weight changes in HSC-2 derived tumor bearing mice during medication periods. Significant differences were not observed within four experimental groups.

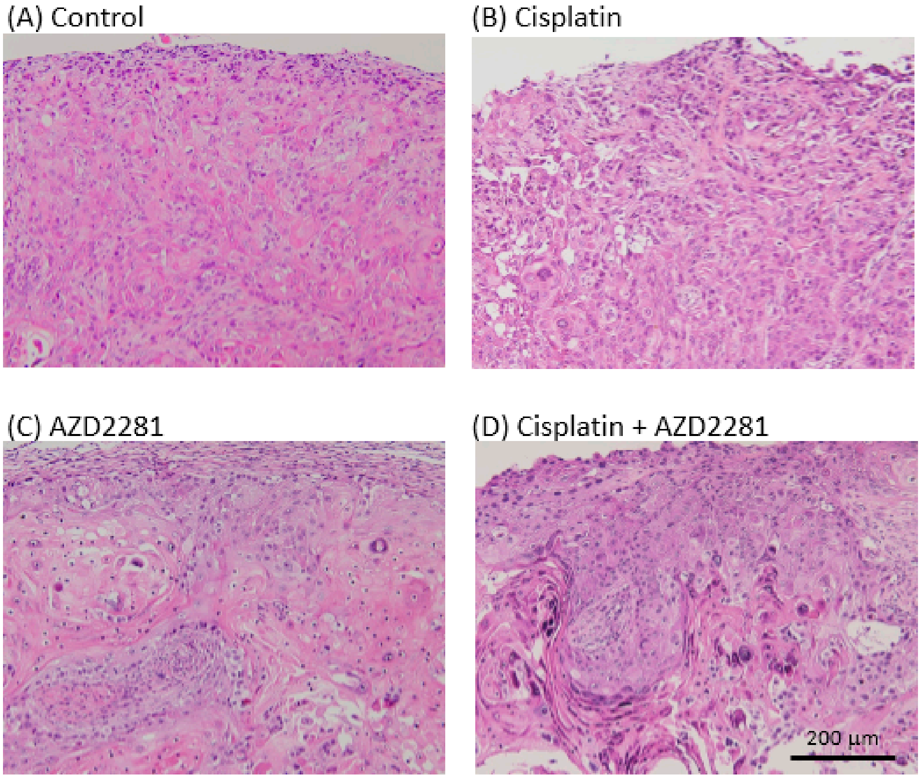

Figure S5. Histological analysis of representative xenografted tumors in control (**A**); cisplatin (**B**); AZD2281 (**C**); and combination treatment (**D**) groups. Scale bar, 200 μm.
